# Atypical Cadherin Fat2 is Involved in Axogenesis of Cerebellar Granule Cells in Zebrafish

**DOI:** 10.1111/dgd.70060

**Published:** 2026-06-09

**Authors:** Jui Chun Wang, Miki Takeuchi, Ryuji Dohaku, Seok‐Hyung Kim, Lilianna Solnica‐Krezel, Takashi Shimizu, Masahiko Hibi

**Affiliations:** ^1^ Department of Biological Science, Graduate School of Science Nagoya University Nagoya Japan; ^2^ Department of Developmental Biology Washington University School of Medicine St. Louis Missouri USA

## Abstract

Fat2 is an atypical cadherin with a large extracellular domain containing multiple cadherin repeats. Although members of the Fat family have been implicated in organ development, the role of Fat2 in neural development remains poorly understood. Here, we investigated the function of Fat2 in zebrafish, focusing on its involvement in the development of cerebellar granule cells. Granule cells in the rostromedial cerebellum project their axons, known as parallel fibers, to Purkinje cell dendrites. In contrast, granule cells in the caudolateral cerebellum project their axons to Purkinje cells and further extend them caudally to innervate crest cells, Purkinje‐like neurons located in the dorsal hindbrain region called the medio‐octaval lateralis. We found that in *fat2* mutants, axons of caudolateral cerebellar granule cells projecting to crest cells exhibit abnormal branching and frequently overshoot their targets. These results indicate that Fat2 is required for proper axon formation in at least a subset of cerebellar granule cells.

## Graphical abstract

Granule cells are the most abundant neurons in the vertebrate brain. The atypical cadherin gene *fat2* is specifically expressed in cerebellar granule cells. In wild‐type zebrafish larvae, granule cells in the caudolateral cerebellum project their axons straight to Purkinje cells (PCs) and further extend them caudally to innervate crest cells (Crs), Purkinje‐like neurons located in the dorsal hindbrain region called the medio‐octaval lateralis. In contrast, in *fat2* mutants, the axons of caudolateral cerebellar granule cells projecting to crest cells show abnormal branching and frequently overshoot their targets.

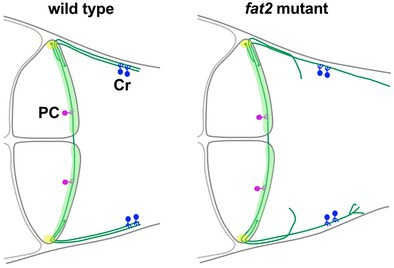

## 1 Introduction

**FIGURE 1:**
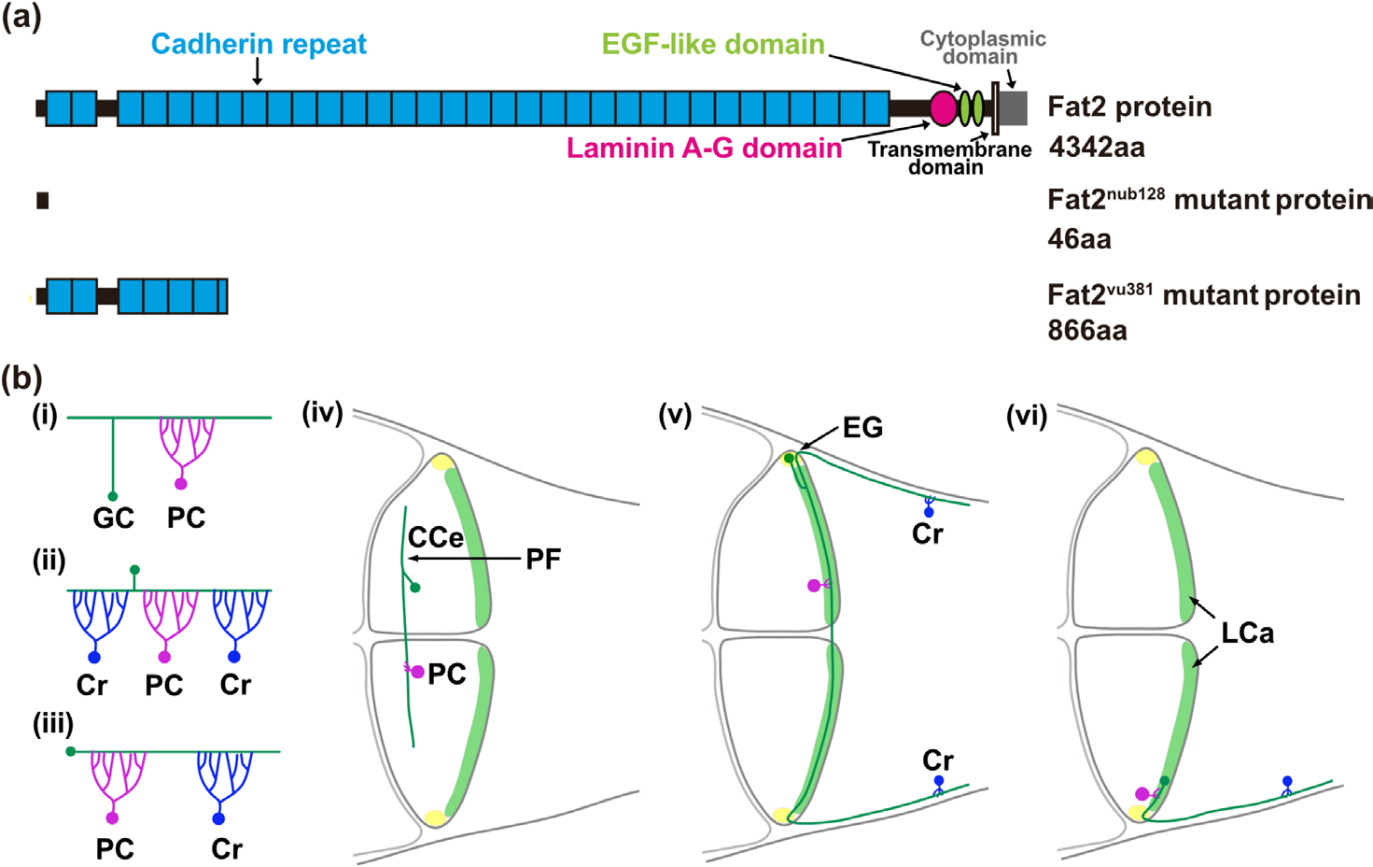
Fat2 protein structure and anatomy of the zebrafish cerebellum. (a) Schematic representation of the domain organization of wild‐type and mutant Fat2 proteins. The wild‐type Fat2 protein consists of 4342 amino acids. The *fat2* ^*nub128*^ allele carries a frameshift mutation and encodes a 46‐amino acid peptide, of which 43 amino acids are derived from the Fat2 and the remaining 3 are unrelated. In contrast, the *fat2* ^*vu381*^ allele carries a nonsense mutation and encodes a truncated protein of 866 amino acids. (b) Schematic illustration of the anatomy of the zebrafish larval cerebellum and its neural circuits, showing axonal trajectories of granule cells in the cerebellar corpus (CCe) (i, iv), granule cells in the eminentia granularis (EG) (ii, v), and granule cells in the lobus caudalis (LCa) (iii, vi), together with a dorsal view of the cerebellum. Green dots indicate granule cells (GCs), magenta dots indicate Purkinje cells (PCs), and blue dots indicate crest cells (Crs). Parallel fibers (PFs) are shown in panel (iv).

Fat‐family proteins are evolutionarily conserved atypical cadherin‐family transmembrane proteins. These single‐pass transmembrane proteins contain numerous cadherin repeats, epidermal growth factor (EGF) domains, and a laminin A–G domain in their giant extracellular domain (Figure 1a) (Tanoue and Takeichi 2005). In *Drosophila*, Fat interacts in trans with another atypical cadherin family member, Dachsous (Dchs). Fat regulates tissue growth via the Hippo pathway, as well as planar cell polarity (PCP) in wing discs and ovarian follicles, and mitochondrial metabolism (reviewed in Kasiah and Mcneill 2023; Sharma and Mcneill 2013; Sadeqzadeh et al. 2014).

There are four members of Fat cadherins in vertebrates, Fat1–Fat4, which are expressed in many tissues and organs during development. Mutations in genes encoding Fat cadherins affect growth and tissue organization. *Fat1*‐deficient mice display a lack of glomerular slits in the kidney (Ciani et al. 2003), eye deformities (Sugiyama et al. 2015), muscle malformations (Caruso et al. 2013; Hou and Sibinga 2009), and neural tube closure defects (Badouel et al. 2015). *Fat4*‐deficient mice show small, cystic kidneys (Bagherie‐Lachidan et al. 2015; Zhang et al. 2019), broadening of the neural tube (Badouel et al. 2015), spine abnormalities (Kuta et al. 2016; Mao et al. 2016), and brain malformations (Cappello et al. 2013; Ishiuchi et al. 2009). Some of these phenotypes can be explained by defects in Hippo or PCP signaling (Kasiah and Mcneill 2023; Sharma and Mcneill 2013; Sadeqzadeh et al. 2014). *Fat3* is specifically expressed in the nervous system (Nagae et al. 2007) and controls cell migration and neurite retraction through regulation of the cytoskeleton in the retina (Krol et al. 2016; Aviles et al. 2022). Compared to Fat1, Fat3, and Fat4, much less is known about the function of Fat2 in organ development. In zebrafish, only limited studies have addressed the functions of Fat family proteins, and the role of Fat2 remains largely unexplored.

The extracellular domain of Fat2 encompasses 33 cadherin repeats, two EGF domains, and one laminin A–G domain, but the intracellular domain of Fat2 differs from those of Fat1, Fat3, and Fat4 in both mammals and zebrafish (Tanoue and Takeichi 2005) (Figure 1a). In rats, *Fat2* mRNA is specifically expressed in granule cells of the cerebellum in the central nervous system, and FAT2 protein is localized to granule cell axons (Nakayama et al. 2002). Homophilic interactions between FAT2 molecules in trans mediate cell–cell adhesion when expressed in mouse L cells (Nakayama et al. 2002). An interaction between FAT2 and CBLN1, a secreted molecule expressed in granule cells, has also been reported in mice and suggested to be involved in synapse formation between granule cells and Purkinje cells (Wang, Pu, et al. 2025). However, the role of Fat2 in neurite formation has not yet been examined. Here, we investigated this role in zebrafish cerebellar neural circuits.

The zebrafish cerebellum has a simpler lobular structure than that of mammals, but cerebellar circuits are largely conserved between mammals and zebrafish, although some differences exist (Hibi et al. 2017; Hibi and Shimizu 2012). As in mammals, granule cells are located in the granular layer, which lies internally, and extend their axons dorsally and bifurcate to form parallel fibers that synapse onto Purkinje cells in the rostromedial cerebellum (valvula cerebelli [Va] and corpus cerebelli [CCe]) (Figure 1bi,iv). In contrast, in the caudolateral cerebellum (lobus caudalis cerebelli [LCa] and eminentia granularis [EG]), granule cells are located superficially and send their axons to Purkinje cells in the cerebellum, and then further extend them caudally to innervate the dendrites of crest cells, Purkinje‐like cells whose somata are located in the medio‐octaval lateralis [MON] (Takeuchi, Yamaguchi, et al. 2015) (Figure 1bii,iii,v,vi). Granule cells are also present in the torus longitudinalis [TL] of the midbrain and send their axons to type I neurons in the optic tectum, forming a cerebellum‐like structure (Bae et al. 2009; Hibi and Shimizu 2012). Circuits comprising granule cells and crest cells are involved in processing vestibular and lateral line information for motor control (Nieuwenhuys et al. 1998). Developmental mechanisms controlling the formation of granule cell axons that project to crest cells are not well understood. We previously reported that granule cell axons projecting to crest cells exhibit abnormal branching in zebrafish mutants for the type IV collagen proteins Col4a5 and Col4a6, components of the basement membrane surrounding granule cell axons (Takeuchi, Yamaguchi, et al. 2015), indicating a role for environmental factors in proper axogenesis of granule cells. However, the intrinsic mechanisms by which granule cell–expressed proteins regulate axogenesis remain elusive.

We previously found that *fat2* is also expressed in cerebellar granule cells in zebrafish larvae (Takeuchi, Yamaguchi, et al. 2017). The zebrafish provides several advantages for studying cerebellar development, including its genetic accessibility, optical transparency, and relatively simple but conserved neural circuitry. In this study, we investigated the role of *fat2* in granule cell axogenesis by generating and analyzing zebrafish *fat2* mutants.

## 2 Materials and Methods

### 2.1 Ethics Declarations

The animal work in this study was approved by the Nagoya University Animal Experiment Committee and was conducted in accordance with the Regulation on Animal Experiments of Nagoya University.

### 2.2 Zebrafish Strains

Wild‐type zebrafish with the Oregon AB genetic background were used. The following transgenic fish lines were used: *Tg(cbln12:Venus)nub24Tg* (Dohaku et al. 2019), *Et(hsp70l:GAL4FFDMC)nkhspGFFDMC90AEt* (Takeuchi, Matsuda, et al. 2015), and *Tg(10xUAS:LIFEACT‐mCherry)chi4Tg* (Mizoguchi et al. 2016).

### 2.3 Generation of *fat2* Mutant by CRISPR/Cas9

The *fat2* ^*nub128*^ mutant allele was generated using the CRISPR/Cas9 method as described previously (Takeuchi, Inoue, et al. 2017). The oligonucleotides 5′‐TAGGCAGAGTTCTCATAAATGC‐3′ and 5′‐AAACGCATTTATGAGAACTCTG‐3′ were annealed and subcloned into the pT7‐gRNA vector (Jao et al. 2013). The target sequence was 5′‐CAGAGTTCTCATAAATGCTGG‐3′, with the PAM sequence underlined. gRNA was synthesized from BamHI‐digested pT7‐gRNA plasmid using T7 RNA polymerase (Promega) and co‐injected with capped Cas9 mRNA. In the F1 generation, a mutant harboring a 32‐bp insertion in exon 1 of *fat2* was isolated.

### 2.4 TILLING Mutant Line

The *fat2* ^*vu381*^ mutant allele was generated by the Targeting Induced Local Lesions IN Genomes (TILLING) method (Kim et al. 2011; Till et al. 2003). In the *fat2* ^*vu381*^ allele, a nonsense mutation converts the CAG codon encoding glutamine at amino acid position 867 to the stop codon TAG.

### 2.5 Genotyping

Genotyping of the *fat2* ^*nub128*^ allele was performed by PCR using the primers 5′‐GCTCTTTGCTGAGGTTCACC‐3′ and 5′‐CCAGAAGGGATCAGTCAGTA‐3′, and mutant alleles were distinguished from wild‐type alleles based on the size difference of PCR products by polyacrylamide gel electrophoresis. Genotyping of the *fat2* ^*vu381*^ allele was performed by PCR using the primers 5′‐TATTCGCTGTTTACCTTCACAGATTTGATT‐3′ and 5′‐ATGAGAAAAGTTGTGGATCCGGTTTG‐3′, followed by digestion with HinfI. PCR products derived from the wild‐type allele, but not those from the *fat2* ^*vu381*^ mutant allele, were digested by HinfI, and genotypes were determined by agarose gel electrophoresis.

### 2.6 Fish Maintenance

Zebrafish were maintained at 28°C under a 14‐h light/10‐h dark cycle. Embryos and larvae were raised in embryonic medium (Westerfield 2000). For immunohistochemistry and whole‐mount in situ hybridization, larvae were treated with 0.003% 1‐phenyl‐2‐thiourea (PTU; Nacalai Tesque, 27,429–22) to inhibit pigmentation.

### 2.7 In Situ Hybridization

Whole‐mount in situ hybridization was performed as previously described (Bae et al. 2009). The expression of *fat2* has previously been reported (Takeuchi, Yamaguchi, et al. 2017). cDNA fragments of *dchs1a*, *dchs1b*, and *dchs2* were amplified by reverse transcription (RT)‐PCR from RNA of zebrafish larvae using the primers listed below and were subcloned into pTAC‐2 (BioDynamics Laboratory Inc.) The primers used were 5′‐GGAAGGACGATAATGGATCC‐3′ and 5′‐CCTTGTGTAGTGAGCTGATC‐3′ for *dchs1a*; 5′‐GCTAAGGATGCTGTTGGTGG‐3′ and 5′‐CCAACATTGCCTTCTTTGATC‐3′ for *dchs1b*; and 5′‐CGAGGAAGAGACAGCAGAG‐3′ and 5′‐CGATCTCTATAAGGCATTCTG‐3′ for *dchs2*. Digoxigenin (DIG)‐labeled riboprobes were generated from linearized plasmids using SP6 RNA polymerase (Promega). Larvae were hybridized with DIG‐labeled probes overnight at 55°C and incubated overnight with a 1:5000 dilution of alkaline phosphatase–conjugated anti‐DIG Fab fragment (Roche, 11093274910) at 4°C. BM Purple AP substrate (Roche, 11442074001) was used for color development. Images were acquired using an Axio Imager microscope equipped with an AxioCam CCD camera.

### 2.8 Immunostaining

Larvae were immunostained as previously described (Bae et al. 2009; Wang, Shimizu, and Hibi 2025). For immunostaining, anti‐Vglut1 (1:500, rabbit polyclonal), anti‐parvalbumin 7 (Pvalb7, 1:1000, mouse ascites) (Bae et al. 2009), anti‐Neurod1 (1:500, mouse ascites) (Kani et al. 2010), anti‐GFP (for Venus, 1:1000, rat; Nacalai Tesque, 04404‐84), and anti‐DsRed (recognizing mCherry, 1:1000, rabbit; Clontech Laboratories, 632496) antibodies were used. CF488A goat anti‐mouse IgG (H + L; Biotium, 20018), CF488A goat anti‐rabbit IgG (H + L; Biotium, 20012), CF488A goat anti‐rat IgG (H + L; Biotium, 20023), CF568A goat anti‐rabbit IgG (H + L; Biotium, 20101), and CF568A goat anti‐mouse IgG (H + L; Biotium, 20102) were used as secondary antibodies. Vglut1 and Neurod1 were detected using CF488A‐conjugated secondary antibodies, whereas the signal for Pvalb7 was detected using CF568A‐conjugated secondary antibodies. Fluorescence images were captured using an LSM700 confocal laser scanning microscope. Projection images were constructed from z‐stack sections using the three‐dimensional projection program associated with the microscope (ZEN; Zeiss). Figures were assembled using Adobe Photoshop, Adobe Illustrator, and Affinity Designer with brightness and contrast adjustments applied equally to all images within each figure.

### 2.9 Quantification and Statistics

To quantify marker‐positive cells in larvae, 1‐μm‐thick serial digital z‐stack sections through the entire cerebellum were used. Marker‐positive cells were manually counted, and the total number of positive cells in each larva was used for statistical analysis. Branched axon length was quantified using ZEN software (Zeiss). Axonal branches were manually identified, and their lengths were measured using ZEN software. Individual branches were traced using the line tool with the measurement function. The total length of branched axons per larva was calculated as the sum of all traced segments. Statistical analyses were performed using GraphPad Prism (version 5.1).

### 2.10 AI‐Assisted technologies

ChatGPT (OpenAI) was used solely to assist with language editing of the manuscript. It was not used for data generation, analysis, interpretation, or figure preparation. All AI‐assisted content was reviewed and verified by the authors, who take full responsibility for the integrity and accuracy of the work.

### 2.11 Data Availability

All data supporting the findings of this study are publicly available in the Zenodo repository (https://doi.org/10.5281/zenodo.18480978), including the datasets underlying the analyses and results.

## 3 Results and Discussion

**FIGURE 2:**
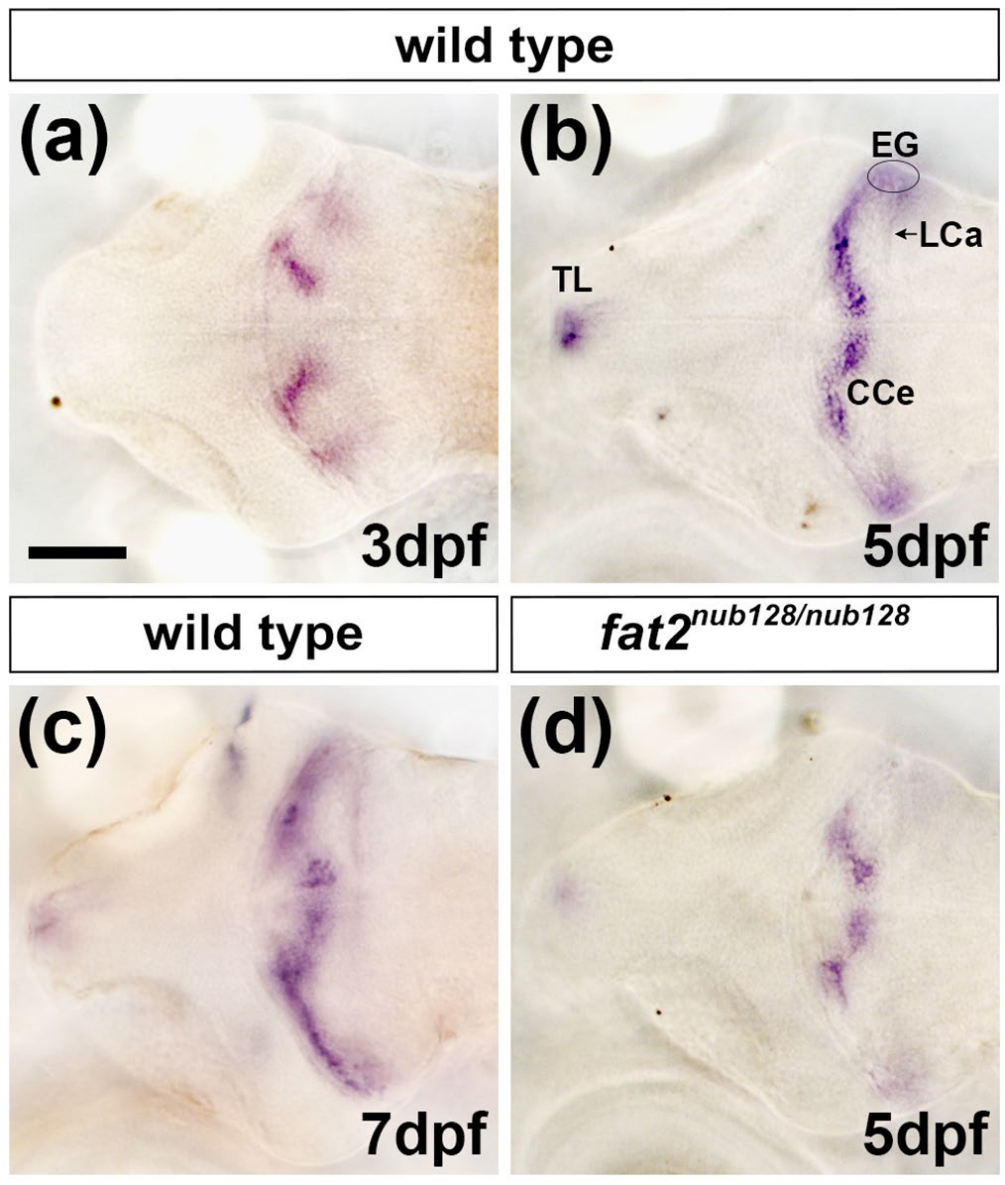
Expression of *fat2* in wild‐type and mutant zebrafish larvae. (a–d) Whole‐mount in situ hybridization showing expression of *fat2* mRNA at (a) 3 dpf, (b) 5 dpf, and (c) 7 dpf in wild‐type larvae, and (d) at 5 dpf in *fat2* ^*nub128/nub128*^ mutant larvae, detected using a digoxigenin‐labeled antisense probe (*n* = 5). Dorsal views with anterior to the left. In wild‐type larvae, signal is detected in cerebellar granule cells, including the CCe, EG, and LCa. In mutant larvae, signal is reduced in the CCe and nearly absent in the EG and LCa. Scale bar: 100 μm in (a) (applies to a–d). Images are representative of the samples examined, and the observed expression patterns were consistent across all samples (*n* = 5).

Our RNA‐seq analysis at 14 days post fertilization (dpf) revealed that *fat2* is specifically expressed in cerebellar granule cells (Takeuchi, Yamaguchi, et al. 2017). We re‐examined its expression by whole‐mount in situ hybridization. Expression of *fat2* was detected in the cerebellum as early as 3 dpf (Figure 2a). This expression gradually became stronger in the cerebellum from 3 to 7 dpf and was detected strongly in CCe and EG, and weakly in LCa (Figure 2b,c). Expression in the torus longitudinalis (TL) of the midbrain appeared at 5 dpf (Figure 2b). Taken together, these expression patterns indicate that *fat2* is expressed as granule cells differentiate.

To investigate the function of Fat2 in granule cell development, we generated a *fat2* ^*nub128*^ mutant using the CRISPR/Cas9 method and a *fat2* ^*vu381*^ mutant by the TILLING method. Both mutant alleles are predicted to encode premature stop codons. The wild‐type *fat2* gene encodes a protein of 4342 amino acids. The *fat2* ^*nub128*^ mutant allele encodes the N‐terminal 43 amino acids of Fat2, followed by approximately three additional amino acids generated by a frameshift, and lacks all known domains, including the cadherin repeats. This mutation is therefore predicted to produce a severely truncated protein lacking all functional domains and is likely to result in a loss of normal Fat2 function. In contrast, the *fat2* ^*vu381*^ mutant allele encodes an 866 amino acid protein containing six cadherin repeats but lacking the remainder of the protein (Figure 1a). Expression of *fat2* in *fat2* ^*nub128/nub128*^ mutant larvae was strongly reduced compared with wild‐type sibling larvae (Figure 2b,d), suggesting that nonsense‐mediated RNA decay (NMD) occurs in this mutant. NMD is typically triggered when a premature termination codon is located upstream of the final exon–exon junction, leading to degradation of the transcript (Chang et al. 2007; Nagy and Maquat 1998). Consistent with this mechanism, the mutation in *fat2* ^*nub128*^ is located in an early exon and is expected to promote NMD. Similar NMD‐mediated transcript reduction has also been reported in zebrafish (El‐Brolosy et al. 2019). Although these mutants may express a small amount of N‐terminal Fat2 fragments, they are likely to represent null alleles.

**FIGURE 3:**
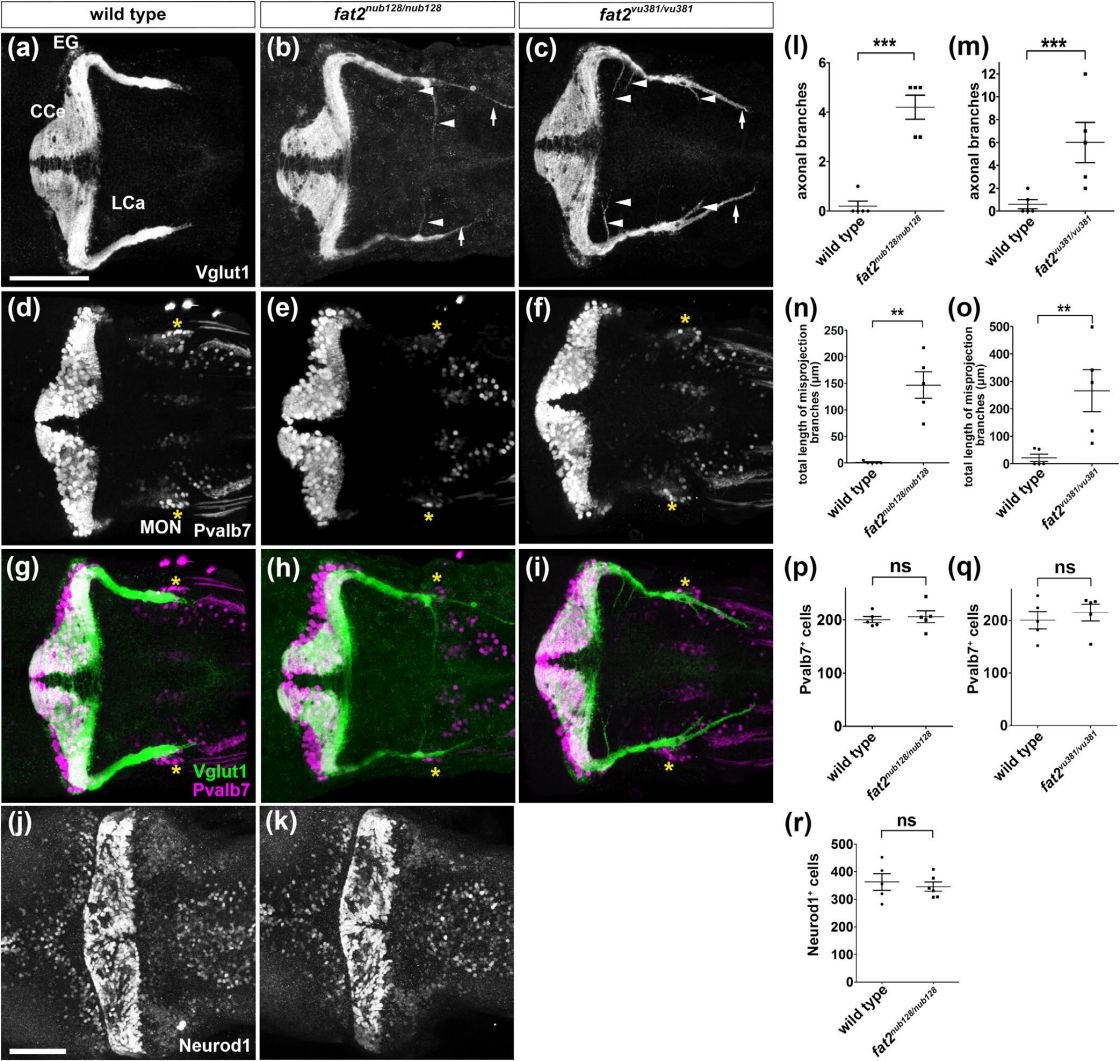
Axonal branching and overextension in *fat2* mutants. (a–k) Expression of Vglut1 (a–c, g–i) and parvalbumin7 (Pvalb7, d–f, g–i) in 5‐dpf wild‐type (*n* = 5), *fat2* ^*nub128/nub128*^ (*n* = 5), and *fat2* ^*vu381/vu381*^ mutant (*n* = 5) larvae. Neurod1 expression in 5‐dpf wild‐type (*n* = 5) and *fat2* ^*nub128/nub128*^ mutant (*n* = 5) larvae (j, k). Signals were detected by immunostaining. Fluorescence signals are shown in grayscale (a–f, j, k) or as two‐color images (g–i; Pvalb7, magenta; Vglut1, green). Dorsal views with anterior to the left. Arrowheads indicate branched axon bundles, and arrows indicate axons extending beyond the crest cell region (overextension). Asterisks indicate crest cells. (l, m) Number of branched axon bundles per larva in *fat2* ^*nub128/nub128*^ (l) and *fat2* ^*vu381/vu381*^ (m) mutants compared with wild‐type larvae. (n, o) Total length of misprojecting axons per larva, measured from branching points to their distal ends, in *fat2* ^*nub128/nub128*^ (n) and *fat2* ^*vu381/vu381*^ (o) mutants compared with wild‐type larvae. (p, q) Number of Pvalb7^+^ Purkinje cells per larva in *fat2* ^*nub128/nub128*^ (p) and *fat2* ^*vu381/vu381*^ (q) mutants compared with wild‐type larvae. (r) Number of Neurod1^+^ granule cells per larva in *fat2* ^*nub128/nub128*^ mutants compared with wild‐type larvae. Welch's *t*‐test. **p < 0.01; ***p < 0.001; ns, not significant. Scale bar: 100 μm in (a) (applies to a–i), and (j) (applies to j, k). Regions of the cerebellum, including CCe, EG, and LCa, are indicated in the images. Images are representative of the samples examined.

We examined the development of cerebellar neurons by immunostaining for Vglut1 (a marker of granule cell axons), parvalbumin 7 (Pvalb7, a marker of Purkinje cells), and Neurod1 (a marker of granule cell nuclei) in early‐stage (5‐dpf) larvae (Figure 3). The expression level of Vglut1 in the cerebellum was not affected in *fat2* ^*nub128/nub128*^ or *fat2* ^*vu381/vu381*^ mutant larvae compared with wild‐type larvae. In contrast, the structure of Vglut1‐positive axons projecting to crest cells was abnormal in these mutants.

In wild‐type larvae, Vglut1‐positive axons from granule cells in the EG and LCa project toward crest cells along the lateral side of the hindbrain and terminate in close proximity to the crest cell region (Figure 3a,d,g). In the mutants, axons exhibit both branching along their trajectory toward crest cells and overextension beyond the crest cell region, as revealed by the relative position of Pvalb7‐positive cells (Figure 3b,c,e,f,h,i). We found that *fat2* ^*nub128/nub128*^ and *fat2* ^*vu381/vu381*^ larvae showed significantly more and longer branched axons compared to wild‐type larvae (Figure 3l–o, Figure S1). These phenotypes were qualitatively comparable between the two mutant alleles. We next quantified axonal overextension by assessing whether axons extended beyond the crest cell region on one or both sides in each larva. Overextension was observed in four of five *fat2* ^*nub128/nub128*^ larvae compared to zero of five wild‐type siblings (Fisher's exact test, *p* = 0.0476), and in five of five *fat2* ^*vu381/vu381*^ larvae compared to one of five siblings (*p* = 0.0476), indicating a significant increase in both mutant alleles. The extent of this phenotype was comparable between the two mutant alleles. Vglut1‐positive axons in the cerebellum (parallel fibers) were not affected in the mutant larvae. The numbers of Pvalb7‐positive Purkinje cells and Neurod1‐positive cells in the cerebellum were not significantly different between wild‐type and *fat2* ^*nub128/nub128*^ larvae (Figure 3d–f,j,k,p,q,r). Pvalb7‐positive crest cells located in the hindbrain were not affected in *fat2* ^*nub128/nub128*^ larvae (marked by asterisks in Figure 3d–i). These data indicate that the absence of Fat2 does not affect the generation of granule cells, Purkinje cells, or crest cells, but rather specifically impairs axogenesis of granule cells in the caudolateral cerebellum.

**FIGURE 4:**
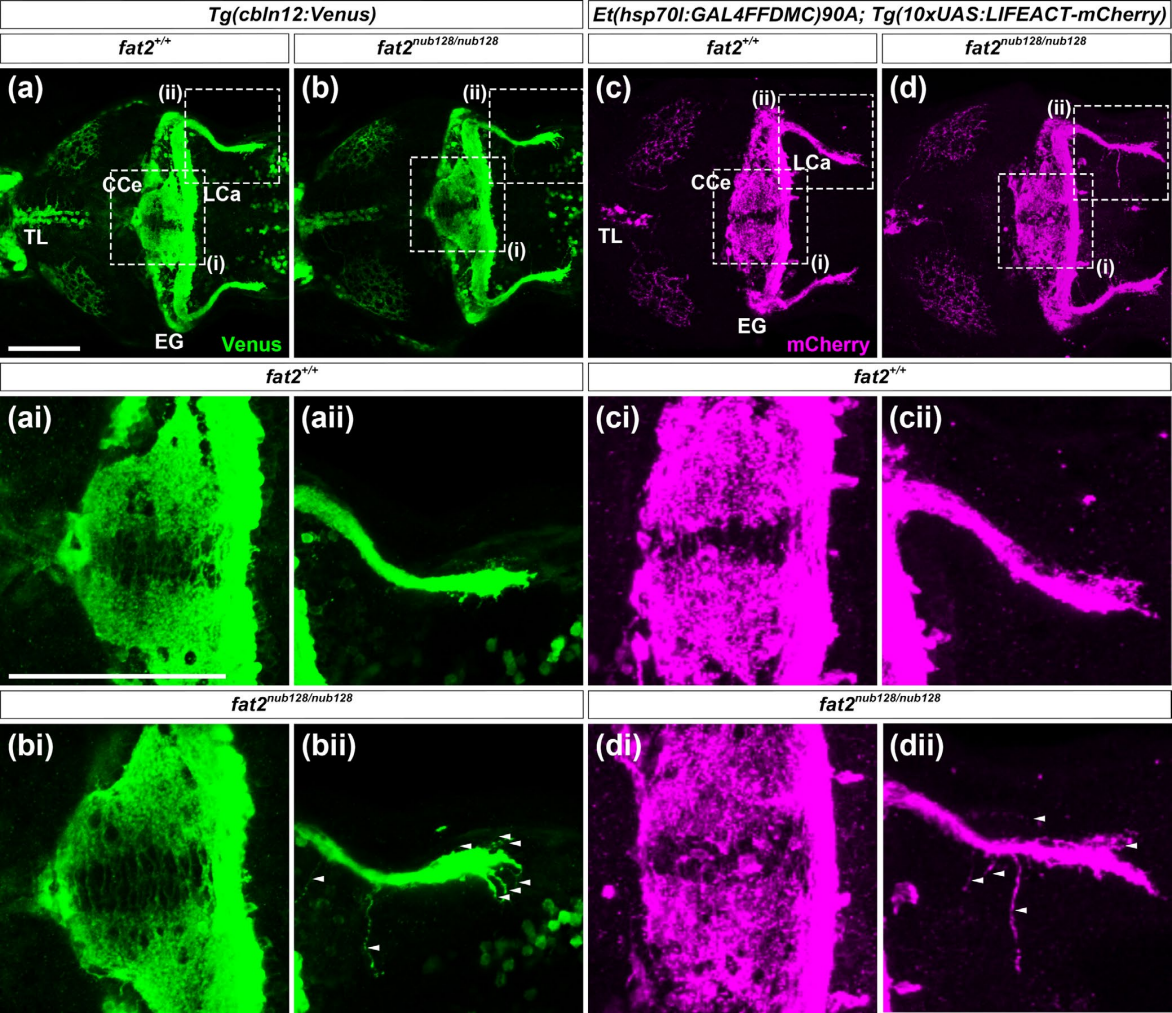
Granule cell morphology and Actin organization in wild‐type and *fat2* mutant larvae. (a–d) Expression of Venus in 5‐dpf *Tg(cbln12:Venus)* larvae in wild‐type (a) and *fat2* ^*nub128/nub128*^ backgrounds (b) (*n* = 5 for each background), and expression of mCherry in 5‐dpf *Et(hsp70l:GAL4FFDMC)nkhspGFFDMC90AEt; Tg(10xUAS:LIFEACT‐mCherry)* larvae in wild‐type (c) and *fat2* ^*nub128/nub128*^ backgrounds (d) (*n* = 5 for each background). Signals were detected by immunostaining. Dorsal views with anterior to the left. (ai–di) Higher magnification views of the corpus cerebelli. (aii–dii) Higher magnification views of granule cell axons in the caudolateral lobes. Arrowheads indicate branched bundles. Scale bar: 100 μm in (a) (applies to a–d) and (aa) (applies to ai–di and aii–dii). Regions of the cerebellum, including CCe, EG, LCa, and TL, are indicated in the images. Images are representative of the samples examined, and the observed phenotypes were consistent across all samples in each condition (*n* = 5).

Because Vglut1 is localized to presynaptic boutons of granule cell axons, Vglut1 staining may not fully visualize axonal morphology. To examine axonal structures more precisely, we used two transgenic lines (Figure 4). *Tg(cbln12:Venus)* expresses Venus throughout granule cells (Figure 4a,b). *Et(hsp70l:GAL4FFDMC)90A* is an enhancer trap line that expresses a modified yeast transcription factor, GAL4FF, in granule cells (Takeuchi, Matsuda, et al. 2015). When crossed with *Tg(10xUAS:LIFEACT‐mCherry)*, actin fibers in granule cell axons can be visualized (Figure 4c,d). Using both transgenic lines, we observed clear projection of granule cell axons toward the crest cell region in wild‐type larvae (Figure 4aii,cii). In contrast, we found aberrant branching of granule cell axons projecting to crest cells in *fat2* ^*nub128/nub128*^ larvae (Figure 4bii,dii). We also detected abnormal protrusions of growth cones in these mutants (Figure 4bii). Although some protrusions from axonal bundles were observed in the lateral cerebellar area (LCa), their frequency did not differ between wild‐type and mutant larvae (Figure 4ai,bi,ci,di). Furthermore, we did not detect abnormalities in granule cell axons within the cerebellum (parallel fibers, Figure 4ai,bi,ci,di) or in the TL, indicating that the abnormal branching and overshooting phenotypes are specific to granule cell axons projecting to crest cells in early‐stage larvae.

How does Fat2 regulate axon formation in cerebellar granule cells? Several possible mechanisms can be considered. First, Fat2‐mediated signaling from granule cell axons may influence the axonal trajectory. We previously found that mutations in type IV collagen genes disrupt the structure of the basement membrane surrounding axons, alter axonal paths, and cause abnormal axonal branching phenotypes similar to those observed in *fat2* mutants (Takeuchi, Yamaguchi, et al. 2015). This raises the possibility that Fat2 indirectly affects the extracellular environment along axonal pathways. However, because Fat2 is a cell‐adhesion molecule, it is less likely to play a direct role in modifying the extracellular matrix.

Second, Fat2 expressed in granule cell axons may regulate axon formation by mediating adhesion to other cells through interactions with other molecules. Fat‐family proteins are known to associate with Dchs‐family cadherins in both trans and cis configurations (Kasiah and Mcneill 2023; Sharma and Mcneill 2013; Sadeqzadeh et al. 2014). In the cerebellum and anterior hindbrain of early zebrafish larvae, *dchs1a* is strongly expressed, whereas *dchs1b* is weakly expressed and its spatial distribution is not clearly resolved, and *dchs2* expression is not detected (Figure S2). At these stages, *fat2* is expressed in cerebellar granule cells (Figure 2). More specifically, *dchs1a* expression is detected in the posterior and medial regions of the cerebellum, likely corresponding to the cerebellar rhombic lip, and in ventromedial regions of the hindbrain posterior to the cerebellum. If Dchs proteins were involved in the axonal projection of granule cells toward crest cells, their expression would be expected along the axonal trajectory in the anterior hindbrain (lateral regions) or in crest cells. However, *dchs1a* and *dchs1b* expressions are not detected in these regions. Therefore, although the involvement of these molecules cannot be completely excluded, their expression patterns do not align with the trajectories of granule cell axons, making this possibility less likely.

Third, Fat2 may be involved in synapse formation between granule cell axons and their target cells, namely Purkinje cells and crest cells. Failure of granule cell axons to form stable synaptic connections with their targets could result in abnormal axonal trajectories. FAT2 has been shown to interact with CBLN1, and *Fat2*‐deficient mice show reduced integrity of synapses between granule cells and Purkinje cells, suggesting a possible role of FAT2 in synapse formation (Wang, Pu, et al. 2025). Granule cell–Purkinje cell synapse formation critically depends on the interaction between Neurexins (NRXNs) expressed in granule cells and the glutamate receptor GRID2 expressed in Purkinje cells, mediated by CBLN1 (Matsuda et al. 2010; Uemura et al. 2010). However, FAT2 does not appear to substitute for NRXNs as a binding partner in this complex (Uemura et al. 2010), suggesting that it is unlikely to function through this canonical synaptic mechanism. Alternatively, Fat2 may participate in synapse formation with crest cells, thereby influencing axon targeting from granule cells to crest cells. However, abnormal axonal branching in *fat2* mutants is already observed before axons reach crest cells, indicating that synapse‐dependent mechanisms alone cannot fully explain the observed phenotypes.

However, a discrepancy exists between zebrafish and mouse models. In *Fat2*‐deficient mice, Vglut1 signals are reduced throughout the molecular layer, indicating impaired synaptic integrity, whereas such reduction is not observed in zebrafish larvae. One possible explanation is that the structural and functional organization of synapses between granule cells and Purkinje cells differs between species. In zebrafish, granule cell–Purkinje cell synapses may rely predominantly on canonical mechanisms such as the NRXN–CBLN–GRID2 complex, whereas in mice, FAT2 may play an additional role in stabilizing or refining these synaptic connections. In contrast, the prominent axon projection defects observed in zebrafish are largely restricted to granule cells projecting to crest cells. These axons form bundled projections, which may make defects in axon guidance or fasciculation more readily detectable. Although crest cells are not present in mammals, Fat2 is expressed in mouse granule cells, raising the possibility that similar mechanisms may contribute to parallel fiber organization in a more subtle manner. Such defects may not be readily apparent at the light microscopic level but could potentially be revealed by more detailed analyses, such as ultrastructural examination.

Finally, Fat2‐mediated interactions among granule cell axons themselves may contribute to axon formation. Homophilic interactions of FAT2 are known to mediate cell adhesion, and expression of FAT2 in L cells has been shown to promote cell aggregation (Nakayama et al. 2002). Axons of caudolateral granule cells normally form tightly organized fascicles, particularly in projections toward crest cells, where axons converge into tightly organized fascicles. In *fat2* mutants, impaired Fat2‐mediated adhesion may disrupt proper bundle formation, allowing individual axons to defasciculate and exhibit branching. The increased number of protrusions observed in growth cones of mutant axons may reflect reduced adhesion between neighboring neurites. The absence of detectable abnormalities in granule cell axons within the cerebellum may reflect the high density of axons in this region, which could mask subtle defects. Alternatively, additional mechanisms independent of Fat2 may regulate axon positioning within the cerebellum and compensate for the loss of Fat2.

Fat2 possesses an exceptionally large extracellular domain, and homophilic Fat2 interactions may help maintain axonal clusters with appropriate spacing. Such a mechanism may be particularly important for orderly axon formation in densely packed granule cells. Our study therefore identifies a previously unrecognized role of Fat2 in regulating granule cell axon organization during cerebellar development.

## Author Contributions

T.S. and M.H. designed the research. S.‐H.K. and L.S.‐K. created and provided the research samples. J.C.W., M.T., and R.D. performed the research and analyzed the data. J.C.W. and T.S. prepared the figures. J.C.W. and M.H. wrote the original draft. J.C.W. and T.S. reviewed and edited the manuscript.

## Funding

Japan Society for the Promotion of Science (Grant Numbers 25K02283, 23K23894, and 23K05845); NIH grants R01HG002995 (National Human Genome Research Institute) and R35GM118179 (National Institute of General Medical Sciences); Uehara Memorial Foundation.

## Conflicts of Interest

The authors declare no conflicts of interest.

## Supporting information


**Figure S1:** Axonal branching and overextension in *fat2* mutants.
**Figure S2:** Expression of *dchs1a* and *dchs1b*.

## Data Availability

All data supporting the findings of this study are publicly available in the Zenodo repository (https://doi.org/10.5281/zenodo.18480978), including the datasets underlying the analyses and results.
