# Supplementary material for: Atypical Cadherin Fat2 is Involved in Axogenesis of Cerebellar Granule Cells in Zebrafish
Source: Dev Growth Differ. 2026 Jun 9;68(4-5):e70060. doi: 10.1111/dgd.70060 (PMC13249537; doi:10.1111/dgd.70060)
Supplement: Supplementary file 1 — Figure S1: Axonal branching and overextension in fat2 mutants. Figure S2: Expression of dchs1a and dchs1b. [file DGD-68-0-s001.docx]

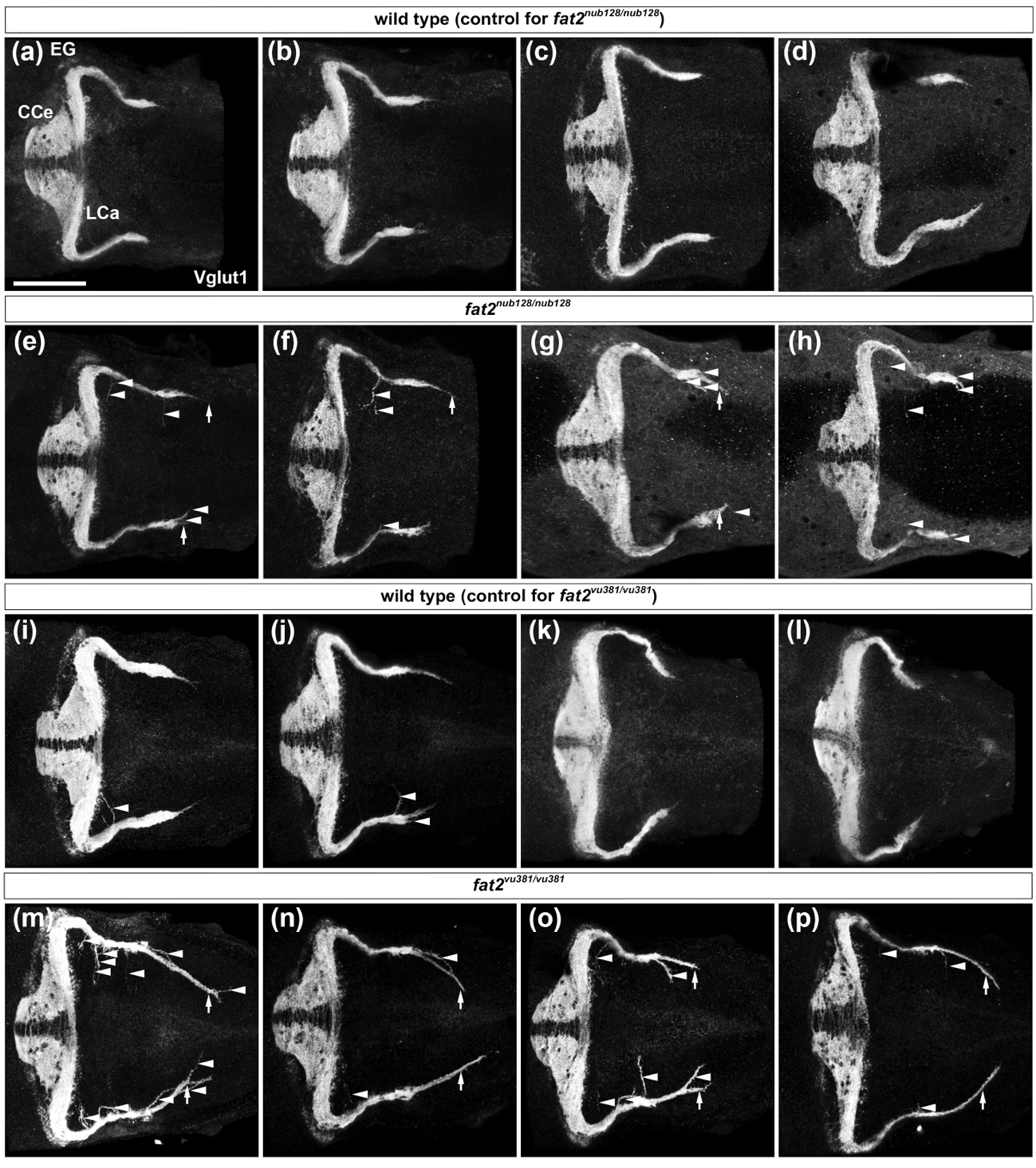


FIGURE S1. Axonal branching and overextension in *fat2* mutants.

(a–h) Expression of Vglut1 in *fat2^nub128/nub128^* mutant (a-d) and wild-type sibling control larvae (e-h). (i–p) Expression of Vglut1 in *fat2^vu381/vu381^* mutant (i-l) and wild-type sibling control larvae (m–p). Signals were detected by immunostaining. Dorsal views with anterior to the left. Arrowheads indicate branched axon bundles, and arrows indicate axons extending beyond the crest cell region (overextension). Scale bar: 100 μm in a (applies to all panels). These images represent additional samples used for quantitative analyses shown in Fig. 3. Representative examples are presented in Fig. 3.


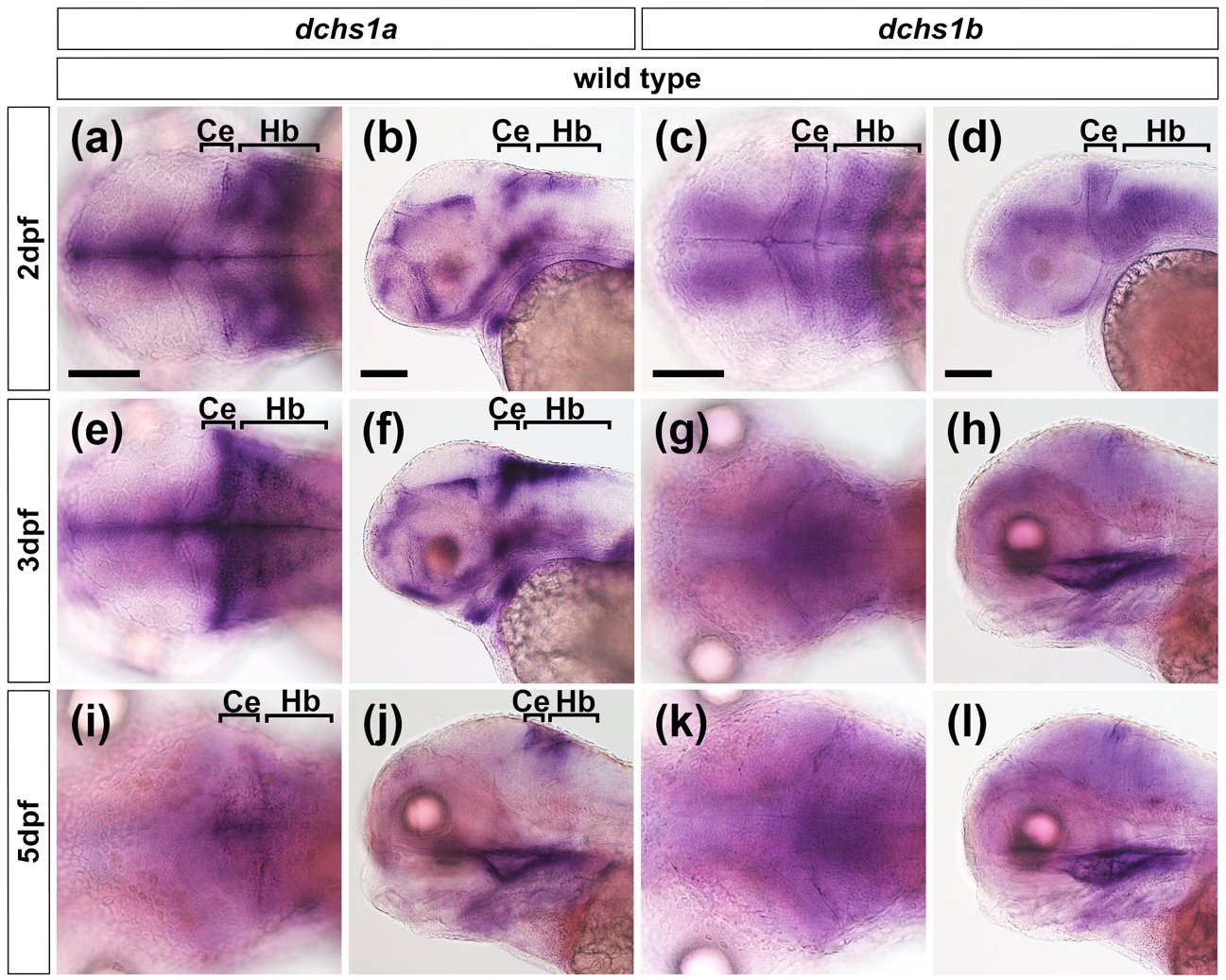


FIGURE S2. Expression of *dchs1a* and *dchs1b*.

(a, b, e, f, i, j) Whole-mount in situ hybridization showing expression of *dchs1a* mRNA in wild-type larvae at 2 dpf (a, b), 3 dpf (e, f), and 5 dpf (i, j), shown in dorsal (a, e, i) and lateral (b, f, j) views with anterior to the left, using a digoxigenin-labeled antisense probe (*n* = 3). (c, d, g, h, k, l) Whole-mount in situ hybridization showing expression of *dchs1b* mRNA in wild-type larvae at 2 dpf (c, d), 3 dpf (g, h), and 5 dpf (k, l), shown in dorsal (c, g, k) and lateral (d, h, l) views with anterior to the left, using a digoxigenin-labeled antisense probe (*n* = 3). Scale bar: 100 μm in a (applies to a, e, i), b (applies to b, f, j), c (applies to c, g, k), and d (applies to d, h, l). Images are representative of the samples examined. *dchs2* expression was not detected in the brain at these stages. Ce, cerebellum; Hb, anterior hindbrain.
